# C3a triggers formation of sub-retinal pigment epithelium deposits via the ubiquitin proteasome pathway

**DOI:** 10.1038/s41598-018-28143-0

**Published:** 2018-06-26

**Authors:** Rosario Fernandez-Godino, Eric A. Pierce

**Affiliations:** Ocular Genomics Institute, Department of Ophthalmology, Massachusetts Eye and Ear Infirmary, Harvard Medical School, Boston, MA 02114 USA

## Abstract

The mechanisms that connect complement system activation and basal deposit formation in early stages of age-related macular degeneration (AMD) are insufficiently understood, which complicates the design of efficient therapies to prevent disease progression. Using human fetal (hf) retinal pigment epithelial (RPE) cells, we have established an *in vitro* model to investigate the effect of complement C3a on RPE cells and its role in the formation of sub-RPE deposits. The results of these studies revealed that C3a produced after C3 activation is sufficient to induce the formation of sub-RPE deposits via complement-driven proteasome inhibition. C3a binds the C3a receptor (C3aR), stimulates deposition of collagens IV and VI underneath the RPE, and impairs the extracellular matrix (ECM) turnover by increased MMP-2 activity, all mediated by downregulation of the ubiquitin proteasome pathway (UPP). The formation of basal deposits can be prevented by the addition of a C3aR antagonist, which restores the UPP activity and ECM turnover. These findings indicate that the cell-based model can be used to test potential therapeutic agents *in vitro*. The data suggest that modulation of C3aR-mediated events could be a therapeutic approach for treatment of early AMD.

## Introduction

The retinal pigment epithelium (RPE) sits on a multilayered extracellular matrix (ECM) called Bruch’s membrane (BrM), the components of which are mostly secreted by the RPE and which acts as a barrier between choriocapillaris and the retina^1,2^. The constant production of the ECM components of BrM is required for the normal function of the eye. Genetic predisposition, environmental factors, and aging promote the excessive deposition of proteins and lipids between the basal lamina of the RPE and BrM, leading to disease^3,4^. These deposits are known as basal deposits and drusen, and appear as yellow spots in the fundus of the eye, and are the first signs of age-related macular degeneration (AMD), which is the most common cause of vision loss in developed countries^5^. Although the mechanisms of deposit formation are not fully understood, the identification of complement risk alleles and the presence of active complement components within the deposits provide compelling evidence that the complement system plays a key role in AMD pathobiology^6–10^. Still, the lack of donor eyes at early stages of AMD and the difficulty in reproducing AMD features in animal models make it challenging to determine the precise role of complement in early stages of disease, when therapies could be administered to prevent disease progression to vision damaging stages of disease, including wet AMD and geographic atrophy. Many theories have been proposed regarding the role of complement in AMD; most of them advocate for the activation of the ultimate complement component, C5, and the membrane-attack complex (MAC), which are present in drusen^11,12^. Unfortunately, anti-complement drugs that target C5 have not shown success in reducing drusen or geographic atrophy in patients with AMD to date^13–15^.

Our group has studied the role of the complement system in the formation of basal deposits over the last decade, focusing on the RPE/BrM pathobiology^16–19^. Using cell-based models, we have demonstrated that the activation of the complement system by the RPE occurs locally, and that abnormalities in the ECM of BrM cause chronic activation of the alternative complement pathway and deposit formation^18,19^. Particularly, we have demonstrated a critical role for C3 in the formation of sub-RPE deposits^16,19,20^. The activation of C3 is common to the three complement pathways (classic, lectin, and alternative). Upon activation by the C3-convertase, C3 is cleaved into its bioactive fragments, C3b and C3a^21^. In the absence of pathogens, C3 can also be activated to C3(H2O) by hydrolysis via tick-over^21,22^. Both C3b and C3(H2O) can bind surfaces, such as foreign cells or ECM and generate a chronic activation of the alternative pathway locally, producing more C3b and C3a^18,21,22^. C3a acts as anaphylatoxin, triggering extravasation of host immune cells upon binding to its receptor, the G-coupled protein C3aR that is a member of the rhodopsin family^21,23^. In the RPE, C3a/C3aR function has been typically associated with endoplasmic reticulum stress, the unfolded protein response, and VEGF secretion^24–26^. We have shown that C3a can trigger the formation of basal deposits by primary mouse RPE cells *in vitro*, although the mechanisms that mediated this response were unknown^19^.

Studies to define the specific role of C3a in early stages of AMD have not been performed to date. Nozaki and colleagues were the first to show an impact of C3a on choroidal neovascularization in a mouse model of wet AMD^26^. More recent studies have associated C3a/C3aR function with oxidative stress and calcium mobilization in the RPE^24,25^. Additionally, C3a has been associated with changes in the proteolytic activity of the proteasome in a mouse model of age-related RPE atrophy^27^. The proteasome is the major cellular non-lysosomal ubiquitin-dependent proteolytic pathway, and it is critical for cell survival^28^. Ubiquitinated proteins are tagged for degradation by one of the three catalytic subunits, ß1, ß2, and ß5^28^. In the retina and RPE, regulation of the ubiquitin proteasome pathway (UPP) can change under stress conditions and aging^29^. Specifically, proteolytic activity of subunit ß5 is diminished by aging in the retina^30^. In line with this study, alterations in the UPP of the RPE have also been associated with AMD, and ubiquitin has been found accumulated in basal laminar deposits and drusen^31–33^.

It is important to highlight that matrix remodeling and degradation is tightly controlled by UPP activity^34^. By modulating the expression and activity of matrix metalloproteinases (MMPs), and their regulators (TIMPs), the proteasome provides a link between the regulation of extracellular proteolytic events and intracellular proteolysis^34^. In our experience, changes in the ECM turnover can lead to the formation of basal deposits by the RPE^2,18^. Thus, we hypothesized that the mechanisms that connect complement activation and basal deposit formation involve dysregulation of the UPP and ECM turnover.

To test this hypothesis, we developed a cell-based model using human fetal (hf) RPE cells that recapitulates the cascade of events that connect complement activation, specifically C3a production and deposit formation by the RPE. The findings demonstrate that C3a binds C3aR expressed in the basolateral membrane of the RPE, causes accumulation of collagens IV and VI underneath the RPE, and diminishes the UPP activity. Proteasome activity ablation results in critical alterations of the ECM turnover by repression of TIMP-3 and excessive activation of MMP-2, which exacerbate the deposition of ECM molecules underneath the RPE and the formation of sub-RPE deposits. Importantly, all these effects are prevented by the addition of a C3aR antagonist. Given the potential immunomodulatory role of C3a in AMD, targeting the C3aR with selective antagonists is a viable therapeutic option, which has demonstrated very promising results in animal models of several human diseases^35^.

## Results

### C3a causes human RPE cells to make basal deposits

**Figure 1:**
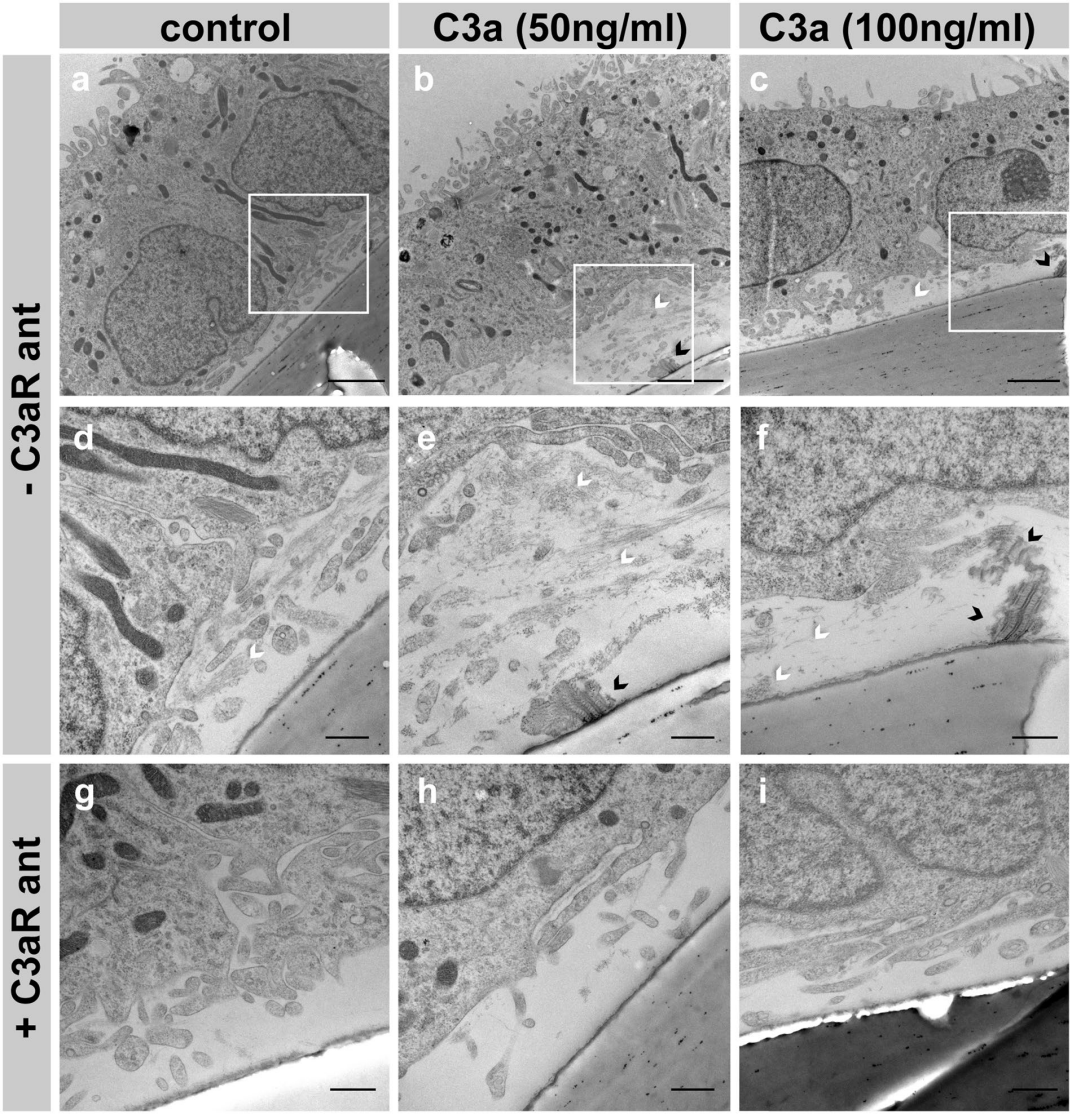
C3a causes abnormal deposition of ECM by hfRPE cells, which is prevented by C3aR antagonist. TEM images show transverse sections of hfRPE cultured on transwells treated for 2 weeks with 0 (**a**,**d**,**g**), 50 (**b**,**e**,**h**) or 100 (**c**,**f**,**i**) ng/ml of C3a in the absence (**a**–**f**) or presence (**g**–**i**) of 10 µM of C3aR antagonist. (**d**), (**e**), and (**f**) are higher magnifications from white squares in (**a**), (**b**), and (**c**) respectively. C3a addition results in the accumulation of collagen fibers (white arrowheads) and wide-spaced collagen (black arrowheads) underneath the RPE cells. The addition of C3aR antagonist prevents the accumulation of sub-RPE deposits caused by C3a (**g**–**i**). Scale bars a–c: 2 µm, d–i: 500 nm.

In an effort to understand the cascade of events that take place upon complement activation in the RPE, we established a system to expose hfRPE cells to different doses of recombinant human C3a for 2 to 4 weeks. We previously demonstrated that C3a causes normal primary mouse RPE cells to make basal deposits *in vitro*, and we asked if human RPE cells would behave similarly^19^. Primary hfRPE cells were isolated and grown on transwells for two weeks followed by 2 to 4 weeks of C3a treatment in the absence of serum. As expected based on the mouse RPE experiments, C3a treatment did not affect the morphology of the RPE monolayer, and transepithelial electrical resistance (TER) was similar among cultures (Supplementary Fig. S1). However, after two weeks of treatment, the hfRPE cells made basal deposits that can be detected by transmission electron microscopy (TEM) (Fig. 1). Deposits were formed by abnormal deposition of ECM fibers. TEM images showed collagen fibers underneath the RPE in all cultures, which increased with increased doses of C3a (Fig. 1a–f). Treatment with C3a also resulted in formation of wide-spaced collagen in some areas underneath the RPE monolayer (Fig. 1e,f). Deposition of ECM fibers was difficult to quantify based on thin transverse sections by TEM.

**Figure 2:**
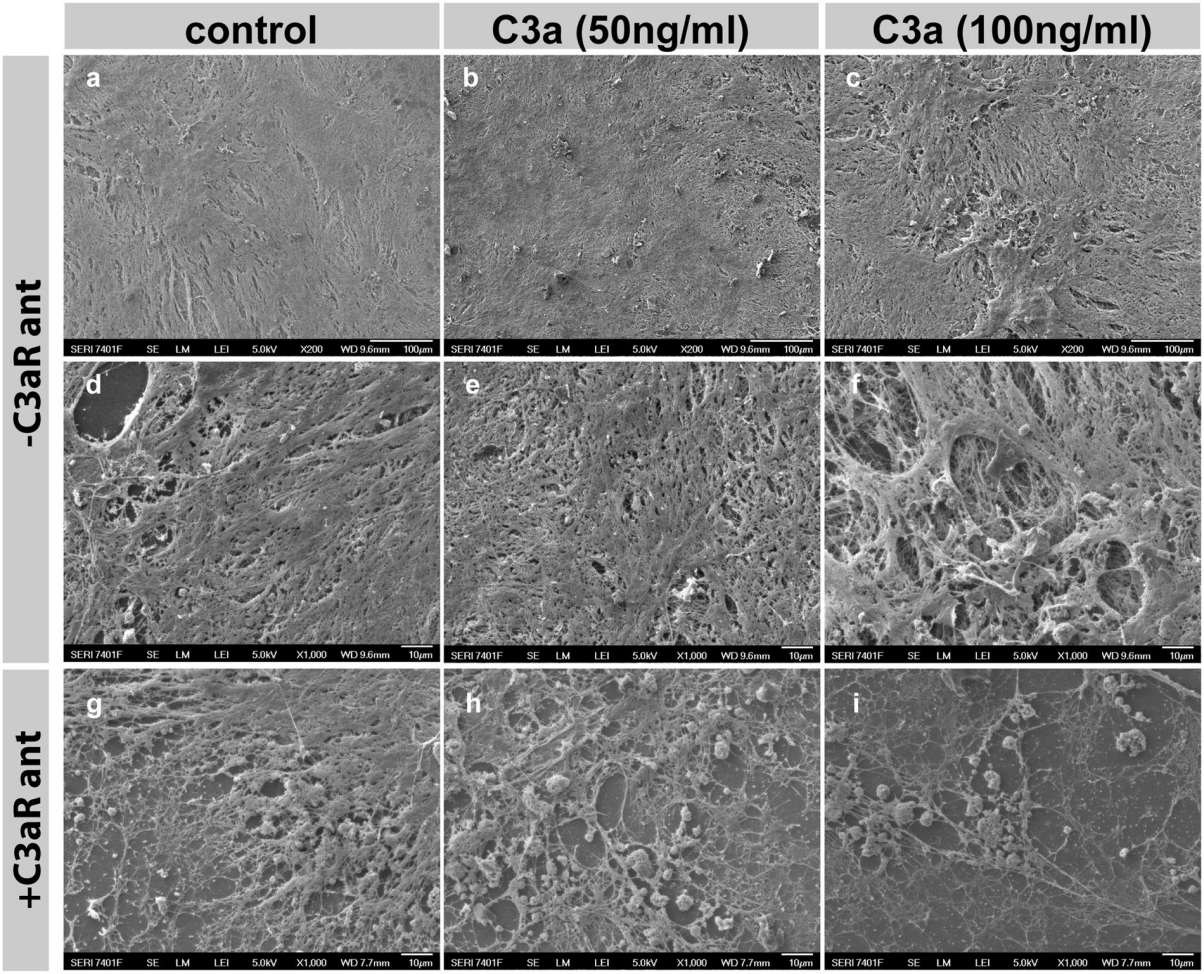
C3aR antagonist prevents formation of basal deposits caused by C3a. SEM images of decellularized transwells show exposed basal deposits made by hfRPE cells treated for 2 weeks with 0 (**a**,**d**,**g**), 50 (**b**,**e**,**h**) or 100 (**c**,**f**,**i**) ng/ml of C3a in the absence (**a**–**f**) or presence (**g**–**i**) of 10 µM of C3aR antagonist. C3a triggers the accumulation of deposits made of ECM fibers underneath the RPE. The abnormal deposition is prevented by the addition of C3aR. Scale bars a–c: 100 µm, d–i: 10 µm.

To determine if the deposition of ECM fibers was homogenous along the transwell, RPE cultures treated with C3a for two weeks were decellularized, and basal deposits were exposed and imaged with scanning electron microscopy (SEM). HfRPE cells treated with C3a deposited thick layers of ECM fibers that coated the surface of the transwell consistently (Fig. 2a–f). Particularly, samples treated with higher doses of C3a accumulated irregular deposits that are portrayed at higher magnification as overlaying coats of dense ECM fibers (Fig. 2f), which extends along the surface of the transwell as a network of fibers with numerous thickened areas that superimpose the ECM underneath. These results are similar to those obtained following treatment of mouse RPE cells with C3a, and by growing hfRPE on abnormal ECM^1,18,19^.

### C3aR antagonist prevents formation of basal deposits caused by C3a

In immune cells, the anaphylatoxin C3a triggers a series of responses by activating its G protein-coupled receptor, C3aR^36^. To test if the effect of C3a was also mediated by its binding to the C3aR in RPE cells and if C3aR blockade could help preventing deposit formation, we cultured hfRPE cells with different doses of recombinant C3a in the presence and absence of trifluoroacetate salt, a specific non-peptide C3aR antagonist, which inhibitory effect on C3aR has been described for many disease models^37–40^. Treatment with C3aR antagonist did not affect the morphology of the RPE monolayer, and TER was similar among cultures (Supplementary Fig. S1).

**Figure 3:**
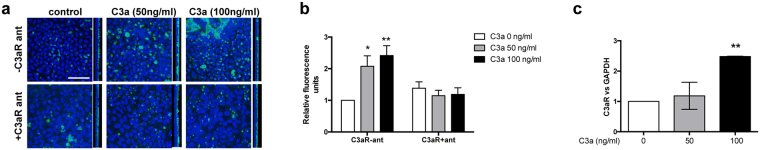
C3a induces overexpression of C3aR in the basolateral membrane of the RPE. (**a**) Immunolabeling with C3aR antibodies of hfRPE cells treated with different doses of C3a (C3aR-ant) or C3a + C3aR antagonist (C3aR + ant) for 2 weeks. Z-stack was built from images taken every 0.5 µm with confocal microscope. 90° projections show that C3aR is expressed on the basal-lateral membrane of RPE. Scale bars 50 µm. (**b**) Average quantification of C3aR fluorescent signal of hfRPE cultures treated with different doses of rhC3a (C3aR-ant) or rhC3a + C3aR antagonist (C3aR + ant). (ANOVA, n = 4/C3aR-ant and n = 4/C3aR + ant treatment. Data represented as mean ± SEM. *p < 0.05, **p < 0.01). (**c**) mRNA levels of C3aR of hfRPE cells treated with different doses of C3a for 24 hours (ANOVA, n = 6. Data presented as mean ± SD. **p < 0.01).

First, immunostaining with antibodies for C3aR demonstrated that it was expressed in the basal-lateral membrane of the RPE (Fig. 3a), and that its expression was augmented with the addition of C3a in a dose-dependent manner (ANOVA, n = 4/treatment. C3a 50 ng/ml: p = 0.0197, C3a 100 ng/ml: p = 0.0022) (Fig. 3b). Based on these results, we hypothesized that blockage of C3aR could have a protective effect for the cells against the increased levels of C3a. We added the non-peptide antagonist of C3aR (SB 290157) along with the recombinant C3a during 2 weeks. Immunolabeling quantification of four cultures per treatment was averaged, and the results indicated no changes in expression of C3aR in cultures treated with C3a regardless the dose if C3aR antagonist was present (Fig. 3).

Additionally, TEM analyses demonstrated that sub-RPE deposits formed in the presence of C3a did not appear when C3aR antagonist was also added to the cultures (Fig. 1g–i). Further, RPE cultures treated for 2 weeks with different doses of C3a in the presence or absence of C3aR antagonist, were decellularized and fixed for SEM. SEM images confirmed that the addition of C3aR antagonist resulted in the formation of a normal network of ECM fibers built on the transwells comparable to the ECM made by control cells (Fig. 2g–i), instead of the accumulation of thick deposits that was produced in the absence of C3aR antagonist (Fig. 2e,f).

### C3a causes increased deposition of collagens IV and VI specifically by RPE cells, which is prevented by the C3aR antagonist

**Figure 4:**
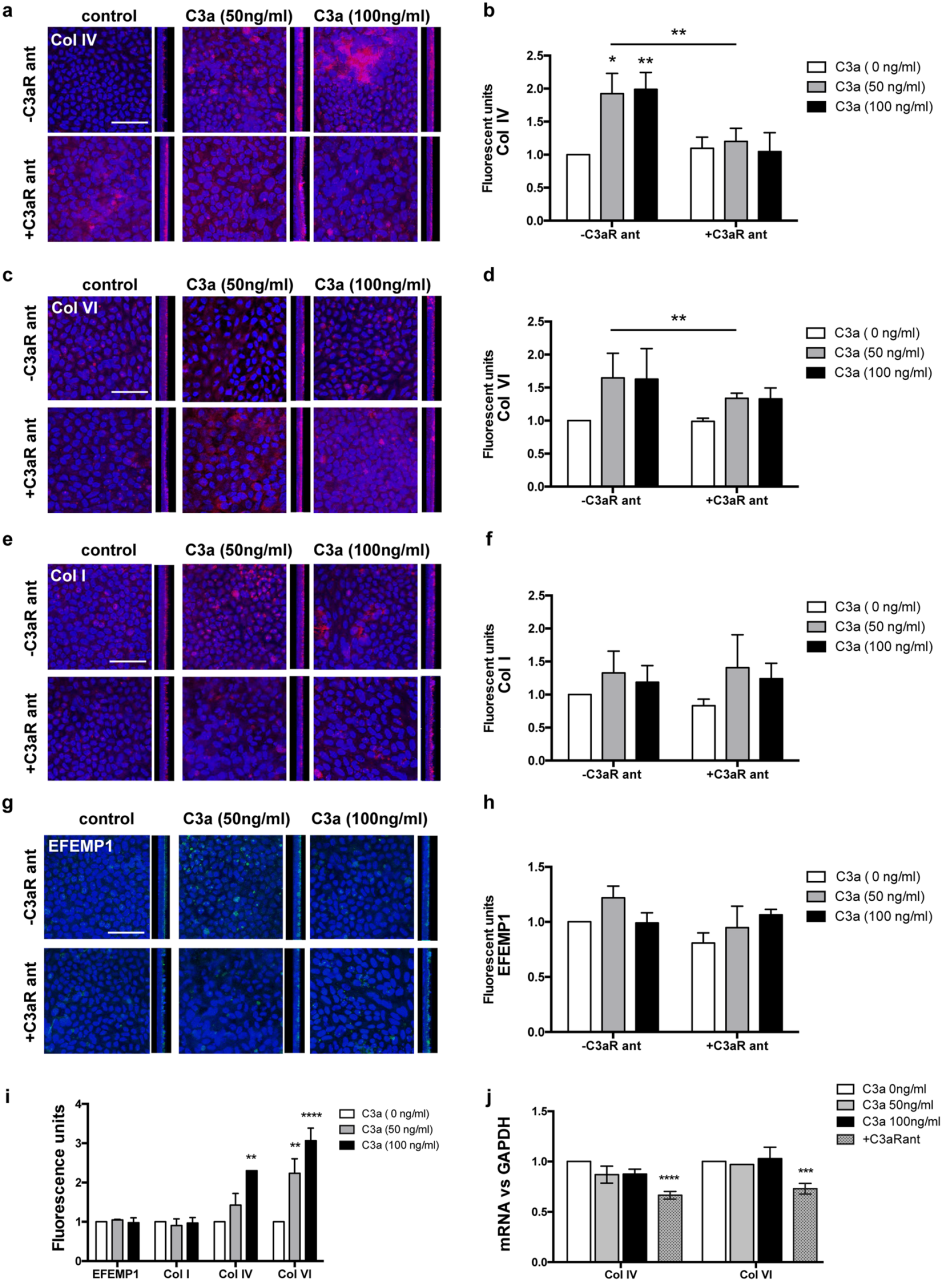
C3a causes specific deposition of Col IV and Col VI underneath the RPE that is prevented by C3aR antagonist. Immunolabeling with antibodies for (**a**) Col IV, (**c**) Col VI, (**e**) Col I, and (**g**) EFEMP1 of hfRPE cells treated with different doses of C3a or C3a + C3aR antagonist for 2 weeks. Images were taken with confocal. Orthogonal views show the basal deposition of the ECM proteins (left: apical, right: basal). Scale bars 50 µm. Average quantification of (**b**) Col IV, (**d**) Col VI, (**f**) Col I, and (**h**) EFEMP1 fluorescent signal of hfRPE cultures treated with different doses of rhC3a (C3aR-ant) or rhC3a plus C3aR antagonist (C3aR + ant) for 2 weeks. (**i**) Average quantification of immunostainings for ECM proteins after 4 weeks of treatment with C3a. (ANOVA. Data represented as mean ± SEM. n = 6/C3a dose *p < 0.05, **p < 0.01). (**j**) mRNA expression of *COL4* and *COL6* normalized to *GAPDH* after treatment with C3a for 2 weeks in the absence and presence (pattern bar) of C3aR antagonist (ANOVA. n = 3/−C3aR ant and n = 3/+ C3aR ant. Data represented as mean ± SD, **p < 0.01, ***p < 0.001, ****p < 0.0001).

To characterize the composition of the sub-RPE deposits formed after the addition of C3a, we performed immunostaining with antibodies for the main components of the ECM of BrM, which we previously found to be major components of sub-RPE deposits *in vitro*^18,19^. The data showed excessive deposition of Col IV after 2 weeks of treatment with C3a, which increased in a dose-dependent manner (ANOVA, n = 6. C3a 50 ng/ml: p = 0.0110, C3a 100 ng/ml: p = 0.0067) (Fig. 4a,b,i). Deposition of Col VI underneath the hfRPE also increased after treatment with C3a (Fig. 4c,d,i), while no changes were observed for Col I (Fig. 4e,f,i) and EFEMP1 (Fig. 4g–i). Expression of other ECM components, such as fibronectin and elastin, was negligible and did not change after 2 weeks of treatment (Supplementary Fig. S2).

The abnormal deposition of collagens increased with time of C3a treatment, showing 2-fold higher amounts of Col IV (ANOVA, C3a 50 ng/ml: p = 0.2424, C3a 100 ng/ml: p = 0.0040) and 3-fold higher amounts of Col VI (ANOVA, C3a 50 ng/ml: p = 0.0014, C3a 100 ng/ml: p < 0.0001) after 4 weeks (Fig. 4i). This appeared to be a post-transcriptional effect, because mRNA levels did not change in the presence of C3a (Fig. 4j).

Based on the fewer amounts of ECM fibers visualized with electron microscope after treatment with C3aR antagonist (Figs 1, 2g–i), we assumed that this molecule prevented either the synthesis or the deposition of collagens.

To investigate the mechanism by which inhibition of C3aR signaling prevents basal deposit formation, we performed immunostaining with antibodies against ECM proteins in hfRPE cells treated with C3a in the presence or absence of C3aR antagonist for two weeks. In parallel, we studied the mRNA levels of collagens in these cultures. In line with the SEM results, these studies demonstrated that the blockage of C3aR prevented the deposition of Col IV and Col VI caused by the addition of C3a (ANOVA, p = 0.0030, p = 0.0084 respectively) (Fig. 4b,d), while other ECM proteins, such as Col I and EFEMP1, did not show a differential expression in the presence or absence of C3a and C3aR antagonist (2-way ANOVA, Col I p = 0.4687, EFEMP1 p = 0.0834) (Fig. 4f,h). qRT-PCR analyses revealed that the addition of C3aR antagonist resulted in decreased mRNA levels of Col IV and Col VI (Fig. 4j) (2-way ANOVA, p < 0.0001 and p = 0.0005, respectively), which implies that the reduced deposition of these proteins underneath the RPE compared to untreated control is due to decreased protein synthesis (Fig. 2g–i).

### No changes in calcium mobilization in RPE cultures were detected after treatment with C3a

**Figure 5:**
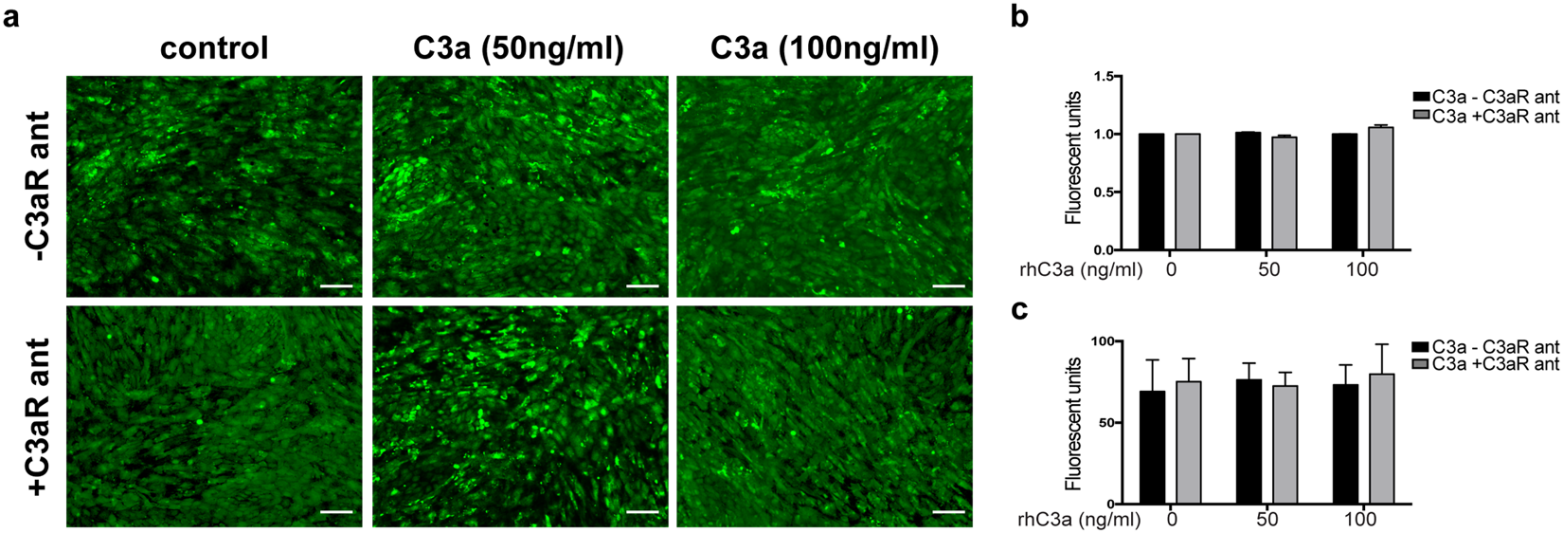
C3a does not make changes in calcium mobilization within the RPE cytosol. (**a**) Confocal fluorescent images of hfRPE cultures treated with different doses of rhC3a only (−C3aR ant) or rhC3a plus C3aR antagonist (+C3aR ant) after incubation with Fluoforte dye®. Scale bars 50 µm. (**b**) Quantification of calcium influx in hfRPE cultures treated with different doses of rhC3a (black bars) or rhC3a plus C3aR antagonist (grey bars), using fluorescent microscope or (**c**) microplate reader. (ANOVA, n = 8/treatment. Data represented as mean ± SD).

Upon C3a binding to the C3aR, intracellular signaling may stimulate calcium influx from the extracellular medium, which may protect the RPE from complement-mediated cell death^41,42^. Accordingly, we tested if the abnormal deposition of ECM proteins could involve calcium mobilization in the cytosol and intracellular stress. We performed two sets of experiments, mimicking acute and chronic activation of the complement system respectively. For the first, we treated hfRPE cells with different doses of C3a for 72 hours, and for the second, treatment was extended for 2 weeks. Quantification of calcium influx was performed using fluorescent microscopy or a microplate reader. Repeated measurements using the Fluoforte® assay did not show differences in the levels of free intracellular calcium between RPE cells treated with different doses of C3a and controls (Fig. 5).

### The ubiquitin proteasome pathway is inhibited upon treatment with C3a in RPE cells

**Figure 6:**
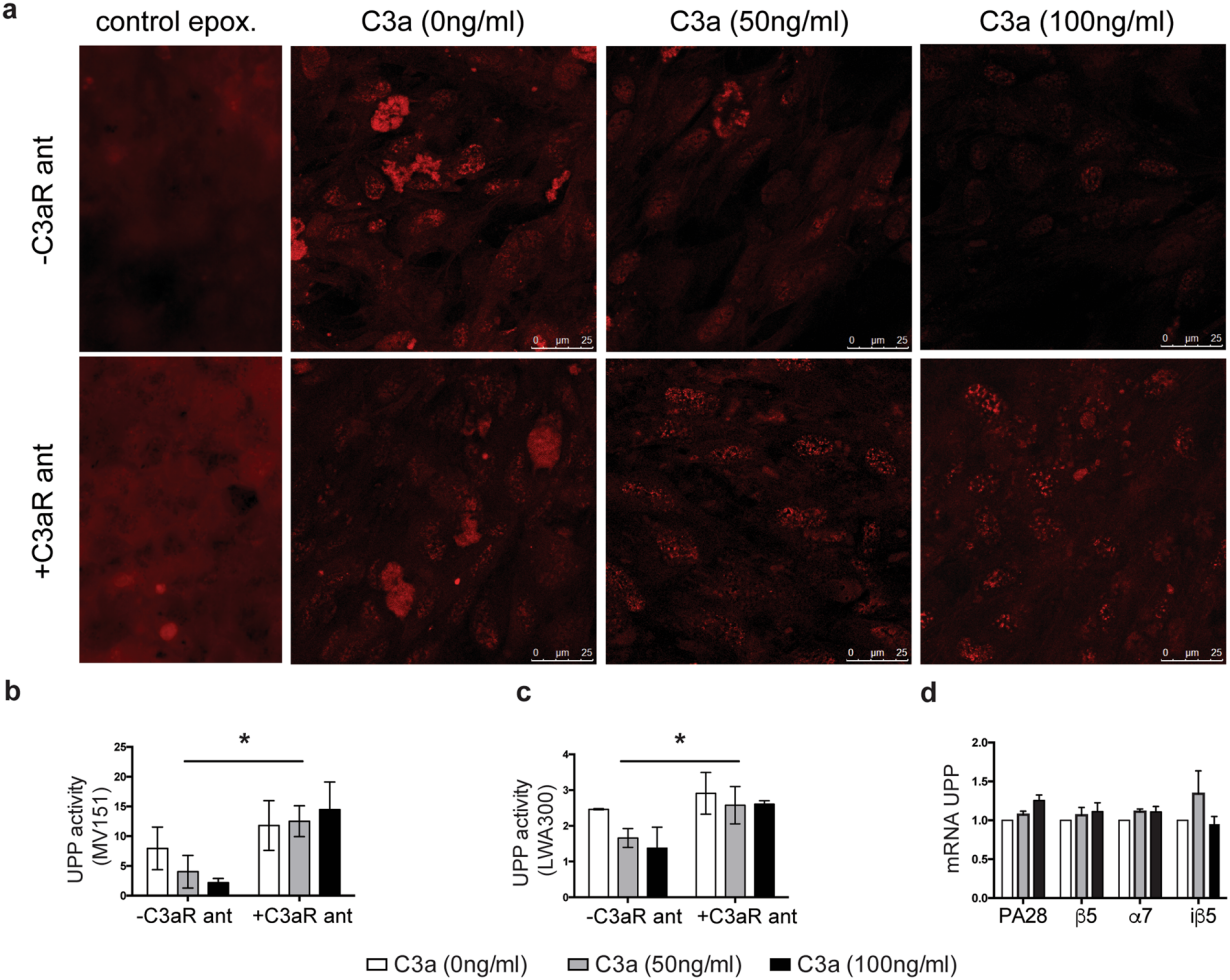
C3a decreases proteasome activity in the RPE, which is restored by the addition of C3aR antagonist. (**a**) Confocal images of UPP overall activity in hfRPE cells stimulated with different doses of C3a with or without C3aR antagonist for 72 hours stained with the probe MV-151. Control epox. = control samples treated overnight with epoxomicin. Scale bars 25 µm. Quantification of UPP overall activity after stimulation with different doses of C3a for (**b**) 2 weeks or (**c**) 72 hours with the probes MV-151 and LWA300, respectively. (**d**) mRNA expression of UPP subunits of hfRPE cells treated for 24 h with different doses of C3a normalized to *GAPDH*. (2-way ANOVA, n = 3/dose. Data represented as mean ± SD. *p < 0.05, **p < 0.01). White bars: control (C3a 0 ng/ml), grey bars (C3a 50 ng/ml), black bars (C3a 100 ng/ml).

After finding that the role played by C3a in the formation of basal deposits was not associated with calcium mobilization, we looked for alternative pathways that have been associated with cellular stress in the RPE, such as UPP components and regulators^33^. To investigate this, we incubated hfRPE cells with different doses of C3a in the presence and absence of C3aR antagonist for 72 hours or 2 weeks, again mimicking acute and chronic activation of the alternative complement pathway. We then treated these cultures with two different fluorescent activity-based proteasome probes (kindly provided by Dr. Overkleeft) to visualize them using confocal microscopy^27,43^. In all cases, the data showed that proteasome activity was decreased after the addition of C3a in a dose-dependent manner (Fig. 6a–c). In order to determine if the downregulation was due to lower levels of expression, we studied the mRNA levels of proteasome-related genes, specifically ß5 subunit and the immune induced ß5i, which are thought to be crucial for the retinal proteasome^30^. mRNA expression for the different proteasome subunits did not change between treated samples and controls (ANOVA, n = 3, p = 0.1593) (Fig. 6d).

Of note, downregulation of the UPP mediated by C3a was prevented in the presence of C3aR antagonist, which allowed hfRPE cells to retain their proteasome activity intact (ANOVA, p = 0.0107 probe MV151, p = 0.0390 probe LWA300, n = 3/dose) (Fig. 6a–c). Thus, complement-driven inhibition of UPP activity in the RPE appears to be mediated by via the C3aR.

### C3a treatment results in increased MMP-2 activity by RPE cells

**Figure 7:**
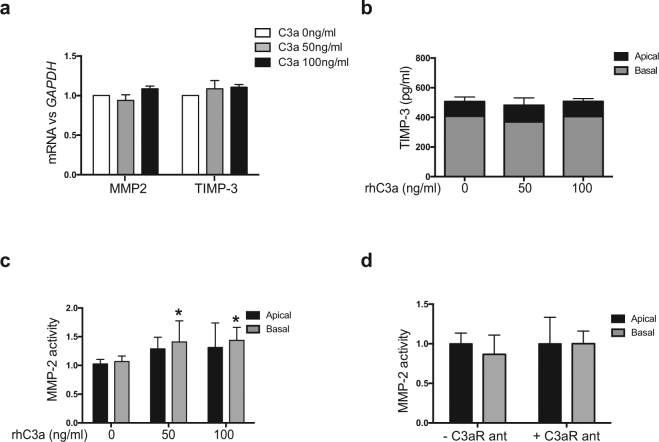
C3a treatment results in increased MMP-2 activity by RPE cells. (**a**) mRNA expression of *MMP-2* and *TIMP-3* measured by qRT-PCR and normalized to *GAPDH* in cultures of hfRPE cells treated with different doses of C3a for two weeks. (**b**) Levels of TIMP-3 measured in conditioned media of the same cultures by ELISA. (**c**) MMP-2 activity measured in apical and basal conditioned media of hfRPE cells treated with different doses of C3a for 2 weeks. (**d**) MMP-2 activity measured in apical and basal conditioned media of hfRPE cells treated with or without C3aR antagonist for 2 weeks (ANOVA, n = 9/treatment. Data presented as mean ± SD. *p < 0.05).

ECM turnover is in part determined by the UPP, which controls the degradation of ECM components and regulators, such as MMP, TIMPs, and collagens^34^. To explore the connection between C3a-mediated dysregulation of the proteasome pathway and altered ECM turnover, we measured the TIMP-3 and MMP-2 activity in conditioned media of hfRPE cells treated with different doses of C3a for 2 weeks. We did not find any differences in the mRNA levels of *TIMP-3* (t-test, p = 0.3746) or presence of TIMP-3 in conditioned basal media (2-way ANOVA, p = 0.0650) (Fig. 7a,b). However, the data showed that MMP-2 activity increased significantly only in the basal media after the addition of C3a in a dose-dependent fashion (2-way ANOVA, n = 9, C3a 50 ng/ml p = 0.0185, and C3a 100 ng/ml p = 0.0106, respectively) (Fig. 7c). Of note, MMP-2 activity did not change with the addition of C3a if cultures were also supplemented with C3aR antagonist (2-way ANOVA, effect of C3aR antagonist: p = 0.1189) (Fig. 7d).

### Inhibition of the proteasome function alters the ECM turnover of the RPE

We next tested the hypothesis that the complement-driven decrease in UPP activity observed was the direct cause for the altered turnover and abnormal deposition of the main components of the basal lamina of the RPE. To do this, we studied the effect of the inhibition of the UPP activity on hfRPE cells using a specific proteasome inhibitor, MG-132^44^. HfRPE cells were passaged and cultured on transwells in serum-free media for a total of four weeks before treatment. After this, 5 µM of the proteasome inhibitor MG-132 was added to the cultures in cycles of 24 hours of treatment followed by 48 hours of wash out for two more weeks.

**Figure 8:**
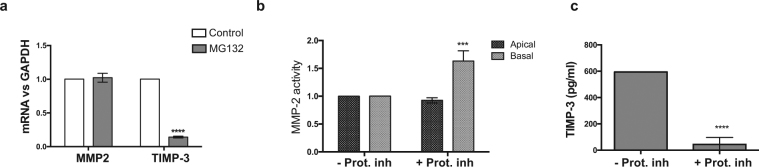
Inhibition of the UPP results in abnormal ECM turnover. (**a**) mRNA expression of MMP-2 and TIMP-3 measured by qRT-PCR and normalized to GAPDH, (**b**) MMP-2 activity measured by zymography, and (**c**) TIMP-3 levels measured by ELISA in conditioned media of hfRPE cells treated with proteasome inhibitor (+Prot inh) for 2 weeks and controls (−Prot inh) (t-test, n = 5. Data presented as mean ± SD. ***p < 0.001, ****p < 0.0001).

To study the impact of proteasome inhibition on the ECM turnover, we analyzed the expression of MMP-2 and TIMP-3^34^. We found that inhibition of proteasome activity decreased TIMP-3 mRNA levels 10-fold (t-test, p < 0.0001, n = 4), and also decreased the amount of TIMP-3 protein secreted by hfRPE cells (ANOVA, p < 0.0001, n = 5) (Fig. 8c). In contrast, no changes were observed in the expression of MMP-2 mRNA (Fig. 8a), although zymography analyses showed that MMP-2 activity increased significantly in basal conditioned media of hfRPE cells treated with proteasome inhibitor (t-test, p = 0.0004, n = 4) (Fig. 8b). Upregulation of MMP-2 activity may be a secondary effect of TIMP-3 downregulation.

Evaluation of deposit formation by hfRPE cells treated with MG-132 was not possible, due to the weakness of the ECM formed by these cells, which resulted in detachment of the monolayer from the transwell upon fixation.

## Discussion

We used an *in vitro* model to investigate the mechanisms that connect the activation of the complement system with the formation of sub-RPE deposits in early stages of AMD. The results presented here demonstrate that C3a, upon binding to C3aR, (1) induces the deposition of collagens IV and VI underneath the RPE, (2) augments collagenase activity by MMP-2 in basal media, (3) and downregulates the proteasome activity, which impacts the ECM turnover via the inhibition of TIMP-3 and excessive MMP-2 activity. The abnormal deposition of collagens and altered proteasome activity, along with the abnormal ECM turnover can be prevented by the addition of a C3aR antagonist. To our knowledge, this is the first report to demonstrate that C3a can stimulate production of basal deposits by normal human RPE cells. Further, the data suggest that modulation of C3aR-mediated events could be a therapeutic approach for treatment of early AMD.

The roles of C3a in innate immunity, upon binding C3aR, include increased vasodilation and vascular permeability. The inflammation model of drusen biogenesis proposes that entrapped material between the RPE and BrM is enough to cause a local inflammatory response, which will cause the activation and recruitment of choroidal dendritic cells and monocytes through the damaged BrM^6,8^. We had previously demonstrated that the activation of the complement system is necessary for the formation of sub-RPE deposits^18–20^. Recently, we have also connected abnormalities in the ECM of BrM with the activation of the alternative complement pathway via tick-over^18^. In all cases as well as in the present study, the central role is played by C3 and does not require further activation of C5 or MAC^21^. These studies offer new insights that may explain the lack of success of AMD drugs targeting complement C5^13–15^. In the present study, the overexpression of C3aR after treatment with C3a could be mediated by cytokines, such as IL-6, the expression of which is upregulated by C3a (Fig. S3). Overexpression of cytokines has been associated with collagen deposition in other disease models such as pulmonary fibrosis and nephropathies^45,46^. Taken together, our data demonstrate that C3 plays a dual role in the formation of basal deposits at early stages of AMD: upon cleavage of C3 by the C3-convertase, C3b and C3a are released. C3b generates chronic activation of the alternative pathway by making more C3-convertase^18^, while C3a binds its G-protein coupled receptor, C3aR, and initiates an intracellular signal transduction that results in the accumulation of sub-RPE deposits.

C3a/C3aR-mediated signaling in the RPE leads to the specific deposition of collagens IV and VI, and wide-spaced collagen, while the levels of Col I remain unchanged. Interestingly, the accumulation of Col IV occurs faster, but the deposition of Col VI is more significant with time. We think that this is due to the progressive accumulation in certain areas of wide-spaced collagen, mainly comprised by Col VI^47^. These results support the accuracy of our model to recapitulate the formation of basal deposits in patients, where Col IV and VI, and typically wide-spaced collagen accumulate underneath the RPE as a first sign of AMD^4,48^.

Of note, C3a causes increased deposition of Col IV and VI without changing mRNA levels, while C3aR blockage seems to decrease collagen deposition via reduced mRNA levels, in the presence or absence of C3a. We think that this difference is due to changes in MMP-2 activity by C3a, which causes abnormal deposition of collagens by altering their turnover. Collagen turnover is performed by collagenases or MMPs and their normal function is key to prevent pathology^2,49^. Using cell-based models, we and others have shown that abnormalities in the MMP-2 activity lead to the accumulation of ECM proteins underneath the RPE and additional inflammation^2,18,50–52^. We have also demonstrated that complement activation may result in increased MMP-2 activity^18^; but these new results reveal that C3a is sufficient to alter the ECM turnover, suggesting a new role for C3a.

The data show that C3a-driven proteasome inhibition results in altered ECM homeostasis by dysregulation of TIMP-3/MMP-2. We think that complement activation makes RPE cells less tolerant to alterations in protein homeostasis due to increased accumulation of ECM protein debris derived from aging or other processes. It is possible that the RPE cells, in an attempt to restore the ECM stability, upregulate the MMP-2 activity. Inhibition of the proteasome via MG-132 has a stronger impact in the ECM turnover than via C3a, because it blocks TIMP-3 at the transcriptional level, which results in 50% increased MMP-2 activity. In both cases, the inhibition of proteasome causes a directional shift in the balance between MMP-2 and TIMP-3. Further studies are needed to reveal the specific mechanisms by which C3a upregulates the activity of MMP-2. Regulation of concentration and turnover of ECM molecules, specially MMPs and TIMPs, has been attributed to the proteasome activity in periprosthetic and tumor microenvironments^34,53^. A reduction in proteasome function has also been associated with aging and other age-related diseases such as Alzheimer’s and Parkinson^54,55^. In the eye, alterations in the proteasome have been described in a mouse model of RPE atrophy exposed to high doses of C3a^27^. We postulate that decreased UPP activity by the RPE results in abnormal accumulation of damaged proteins and lipids, and further dysregulation of inflammatory responses, which are thought to be etiologically related to the formation of drusen^56–58^. In this respect, ubiquitin has been found to accumulate in sub-RPE deposits of AMD patients, suggesting that proteins tagged within the RPE have failed to be degraded by the UPP^31,59^.

The increased deposition of collagens is a C3aR-mediated response and it can be prevented with C3aR antagonist. To highlight, C3aR blockage downregulates collagen expression and results in a thinner layer of ECM fibers deposited underneath the RPE, which can be seen by SEM. This anti-fibrotic effect of C3aR antagonist could have a beneficial effect in the aged eye, as has been shown for other tissues like kidney, lung, and heart^45,46,60^. The addition of C3aR antagonist also restores the ECM homeostasis by stabilizing the MMP-2 activity. These promising results open new avenues to the design of complement-based therapies and suggest that C3aR is a druggable target with potential therapeutic effect to prevent AMD progression if administered at early stages of disease. As a proof of concept, C3aR antagonists have shown therapeutic benefit in a number of rodent models of Alzheimer’s disease and other disorders which pathologies also imply deregulation of ECM turnover and complement activation^60–63^. However, the design of complement-based therapies requires comprehensive understanding of the specific mechanisms that involve complement activation in each individual disease, and given the dual role of C3 in AMD, a combination of C3aR inhibitors with blockage of C3b or C3 activation should be considered^18^.

In summary, C3a-driven proteasome inhibition impacts the formation of basal deposits by altered degradation of collagens and other proteins, which are deposited in excess underneath the RPE. The novel findings reported here reveal important links between complement signaling and the formation of basal deposits in early AMD, highlight new complement functions in the RPE, and demonstrate how excessive complement activation may exacerbate the deposition of ECM proteins via proteasome inhibition. The results provide preliminary data to support consideration of C3aR-inhibition as a new strategy to treat AMD.

## Methods

### Isolation and culture of human fetal RPE cells

Eyes from 16–20 weeks of gestation fetuses were obtained from Advanced Bioscience Resources (Alameda, CA) placed in RPMI on ice, and delivered by an overnight priority delivery service. All tissues were used less than 24 hours after enucleation. All experiments were carried out in strict accordance with institutional, federal, and ARVO guidelines regulating the use of human fetal tissue. All experimental protocols were approved by the Mass Eye and Ear Committee. Primary hfRPE cells were collected as previously described with minor modifications^64,65^. Cells from one eye were resuspended in RPE media + 15% fetal bovine serum (FBS) and seeded on a total of 6 transwells pre-coated with mouse laminin. Confluence after 48 hours was around 30% and media was changed to RPE media + 5% FBS. Serum was removed from the media after 1 week and treatments were added in RPE-serum free media. RPE media was prepared as previously described^64,66^. N1 Medium Supplement 1/100 vol/vol, glutamine 1/100 vol/vol, penicillin-streptomycin 1/100 vol/vol, and nonessential amino acid solution 1/100 vol/vol, hydrocortisone (20 µg/L), taurine (250 mg/L), and triiodo-thyronin (0.013 µg/L) in alpha MEM^64^.

Treatment of hfRPE with recombinant human C3a (rhC3a)(R&D systems, Minneapolis, MN). Different doses of rhC3a (50 ng/ml or 100 ng/ml) diluted in RPE media without FBS were added to the cultures every 72 hours for 2–4 weeks.

Treatment with C3aR antagonist trifluoroacetate salt (SB 290157, Cayman Chemical, Ann Arbor, MI). C3aR antagonist was resuspended in ethanol as indicated by manufacturer and diluted in serum-free RPE media (with or without rhC3a) to a final concentration of 10 µM, which is 50 times higher than the IC50 calculated for this molecule^67^. Cells were treated with C3aR antagonist every 72 hours for 2 weeks.

### Treatment with proteasome inhibitor MG-132 (Sigma Aldrich)

MG-132 was resuspended in DMSO as indicated by manufacturer, and diluted in serum-free RPE media to a final concentration of 5 µM. Due to the high toxicity of this component, hfRPE cells freshly isolated (passage 0) could not be used for these experiments. Therefore, hfRPE cells were expanded for 4 weeks in serum-free RPE media. HfRPE cells at passage 1 were treated for two additional weeks in cycles of 24 hours followed by 48 hours wash in serum-free RPE media. Matching concentration of DMSO was added to controls to rule out side effects due to DMSO toxicity.

### Transmission electron microscopy (TEM)

HfRPE cells on transwells were fixed with 2.5% Glutaraldehyde in 0.1 M sodium cacodylate buffer for a minimum of 2 hours at RT. The inserts were cut into smaller pieces, and post-fixed in 1.0% osmium tetroxide in cacodylate buffer for 1 hour at RT, then rinsed in cacodylate buffer. Insert pieces were then dehydrated through a graded series of ethanols, and placed pre-infiltrated overnight with propylene oxide and Eponate 1:1. Specimens were embedded in Eponate resin. 70 nm sections were cut using a Leica EM UC7 ultramicrotome, collected onto formvar-coated grids, stained with uranyl acetate and Reynold’s lead citrate and examined in a JEOL JEM 1011 transmission electron microscope at 80 kV.

### Decellularization of RPE cultured on transwells

Transwells were decellularized by incubating them with sterile 0.5% Triton X-100 + 20 mM NH4OH in PBS (1.5 ml was added to the bottom chamber and 0.5 ml to the apical chamber) for 5 min at 37 °C, followed by several washes with sterile PBS. Inserts with the exposed ECM were fixed for SEM and immunostaining.

### Scanning electron microscopy (SEM)

Transwell inserts containing exposed ECM after decellularization were fixed with 4% PFA for 10 min and 1% glutaraldehyde for 30 min at RT, followed by critical dehydration and Chromium coating as previously described^18^. Samples were imaged by Field Emission Scanning Electron Microscope (JEOL 7401 F).

### Immunofluorescence of ECM proteins

Transwell inserts containing hfRPE cells were rinsed in PBS, fixed for 10 min in 4% paraformaldehyde (PFA) in PBS followed by fixation in 1% glutaraldehyde for 30 min at room temperature. Inserts were cut into small pieces and blocked with 1% BSA for 30 min at RT. Primary antibodies were incubated overnight at 4 °C. Primary antibodies used were: Col IV (AB6586, Abcam, Cambridge, MA), Col VI (AB6588), Col I (AB34710), FN (AB2413), EFEMP1 (SC33722, Santa Cruz Biotechnology, Santa Cruz, CA), and C3aR (HM2195, Hycult Biotech, Plymouth Meeting, PA). Secondary antibodies labeled with Alexa-488 or Alexa-555 (Life Technologies, Grand Island, NY) were incubated for 1 h at RT. Controls were incubated only with secondary antibody. Sections were mounted with fluoromount G (Electron Microscopy Sciences, Hatfield, PA) and visualized by TCS SP5 II confocal laser scanning microscope (Leica). 90° projections: z-stack was built from images taken every 0.5 µm. Tridimensional 90° projections were performed with ImageJ^68^. Quantification of fluorescent signal was performed by converting z-stacks to 8-bit binary images and measuring the integrated intensity with ImageJ^68^.

### RNA extraction and qRT-PCR

RNA was extracted with RNeasy Mini Kit (Qiagen, Venlo, Netherlands). The quality and quantity of RNA was assessed using the Agilent RNA 6000 Nano Kit Bioanalyzer (Agilent Technologies, Santa Clara, CA). All samples had RIN values higher than 9. cDNA was synthesized using Affinity Script cDNA Synthesis kit (Agilent Technologies, Santa Clara, CA). 10 ng of cDNA, 200 nM of each primer and 10 µl of x Brilliant III Ultra-Fast SYBR Green were combined. Amplification was done in the Stratagene Mx3000P® QPCR system using the following program: 95 °C for 3 s, 40 cycles of 95 °C for 15 s, 60 °C for 20 s followed by melting curve. Each sample was assayed in triplicate. For each experiment, the expression level of the control/untreated sample was used as calibrator. mRNA expression levels were normalized to *GAPDH* as an endogenous reference. Primers used for amplification are the following: *Ubiquitin Proteasome subunits*^27^*: PA28* (F 5′CAGCCCCATGTGGGTGATTATC 3′, R 5′GCTTCTCGAAGTTCTTCAGGATGAT 3′)*; ß5* (F 5′CCTGGAAGGCCAATGCCATAG 3′- R 5′ TTTGCCACCTGACTGAACCACTTC 3′)*; α*7 (F 5′ CCATGATCTGTGGCTGGGATAAG 3′- R 5′GGTCATAGGAATAGCCCCGATC 3′)*; iß5* (F 5′ CTGGAGGCGTTGTCAATATGTACC 3′- R 5′ GCAGCAGGTCACTGACATCTGTAC 3′). *COL6* (F 5′CTCATTCTGCATCCTGGCTTGA3′- R 5′GCCCTGCTGAGGTCTGTGAACA 3′); *COL4* (F 5′AATAACGTGGAGCAAGTGTGC 3′- R 5′GTCTTCCAGGATCTCCGGC3′); *TIMP-3* (F 5′ ACGATGGCAAGATGTACACAGG 3′- R 5′ GGAAGTAACAAAGCAAGGCAGG 3′); *MMP-2* (F 5′ TCTCCTGACATTGACCTTGGC 3′ - R 5′ CAAGGTGCTGGCTGAGTAGATC 3′); *C3aR* (F 5′ ACCAGACAGGACTCGTGGAGACAT - R 5′ GCAGAGAAAGACGCCATTGCTAAAC).

### MMP-2 activity

Was measured in conditioned media of RPE cultures by zymography. Briefly, 10 µl of media were loaded onto Novex 10% gelatin gels (Life Technologies, Grand Island, NY). Zymography assays were then performed per manufacturer’s instructions. Gels were scanned using the Odyssey system (Li-Cor, Lincoln, NE). MMP-2 was identified by molecular weight. Gelatinase activity was quantified using densitometry and the software ImageJ.

### ELISA

Apical and basal conditioned media from cells cultured on transwells and treated with rhC3a were collected every 72 hours and concentrated to equal volumes through 3 kDa Amicon filters (Millipore, Billerica, MA). The fraction over 3 kDa was used to quantify TIMP-3 and IL-6 using ELISA kits from R&D Systems (Minneapolis, MN) following manufacturer’s instructions. All samples were assayed at least per duplicate.

### Ubiquitin Proteasome Activity

The use of high-resolution activity-based fluorescent probes allows the analysis of all proteasome activities in one experiment in living cells, can be used for the identification of the active site peptides and is applicable to clinical samples and can distinguish between constitutive activity and immunoproteasome, which we considered key for this study, given the role of the complement system in proteasome activation^69^. Recent publications show that these activity-based probes are more suitable to measure proteasome activity than other proteomics techniques^70^. UPP activity was detected using the activity-based probes LWA300 (tagged with a green fluorochrome) and MV151 (tagged with a red fluorochrome) kindly donated by Prof. Hermen Overkleeft^71^. These probes are proteasome inhibitors labeled with a fluorescent tag that bind to all the catalytic subunits β1,2,5, and the immune induced β1i,2i,5i. As a negative control, a non-fluorescent proteasome inhibitor probe epoxomicin was used to normalize signal versus residual proteasome activity. Fluorescence of each sample was normalized to the fluorescent background of control samples treated with epoxomicin. hfRPE were seeded on coverslips on 96-well plates and treated with rhC3a with or without C3aR antagonist in RPE media in the absence of serum. After 2 weeks, control samples were incubated with 500 nM of epoxomicin overnight. Next day, all samples (controls and probands) were incubated with 500 nM of LWA300 or MV151 diluted in RPE media for 4 hours at 37 °C. Cells were washed with media and fixed with 50% methanol in PBS 1x for 10 min at RT. Cells were washed twice with fixative solution in order to remove fluorophores stuck to the cell membrane. Coverslips were mounted on slides using fluoromount G and imaged with TCS SP5 II confocal laser scanning microscope (Leica). Fluorescent signal was quantified from images converted to 8-bit binary and measuring the integrated intensity using Image J^68^.

### Calcium Assay Fluoforte

FLUOFORTE® Calcium Assay Kit (Enzo, Farmingdale, NY) was used to measure calcium influx following manufacturer’s instruction. We performed two sets of experiments: (1) 5 × 10^4^ hfRPE were seeded on coverslips on 96-well plates in RPE media +5% FBS for 24 hours. After 24 h serum was removed and cells were cultured for additional 3 days in the absence of serum. Then, cells were treated with rhC3a with or without C3aR antagonist in RPE media for 72 hours. (2) 1 × 10^4^ hfRPE cells were treated with rhC3a with or without C3aR antagonist in RPE media in the absence of serum for 2 weeks. 4 replicates were assayed per treatment per set of experiments (2 for microscope and 2 for microplate reader). After three days or two weeks, medium was removed and 100 µl of Fluoforte dye-loading solution was added per well. Samples were incubated at 37 °C for 45 min and another 15 min at RT. Fluorescent signal was quantified using a fluorescent microscope (FITC spectrum) or in a microplate reader at excitation 485 nm/emission 525 nm per duplicate measured at 2 time-points following manufacturer’s instructions.

## Electronic supplementary material

Supplementary Info.
