# Supplementary material for: C3a triggers formation of sub-retinal pigment epithelium deposits via the ubiquitin proteasome pathway
Source: Sci Rep. 2018 Jun 26;8:9679. doi: 10.1038/s41598-018-28143-0 (PMC6018664; doi:10.1038/s41598-018-28143-0)
Supplement: Supplementary file 1 — Supplementary Info. [file 41598_2018_28143_MOESM1_ESM.pdf]

SUPPLEMENTARY INFORMATION

Title. **C3a triggers formation of sub-retinal pigment epithelium deposits via the ubiquitin proteasome pathway.**

Rosario Fernandez-Godino[1, *], Eric A. Pierce[1].

[1]Ocular Genomics Institute, Department of Ophthalmology, Massachusetts Eye and Ear Infirmary, Harvard Medical School, Boston, MA. 02114 USA.

*Corresponding author: Rosario Fernandez-Godino (rosario_godino@meei.harvard.edu)

243 Charles st. Boston, MA 02114. USA

Tel: 617-573-6787

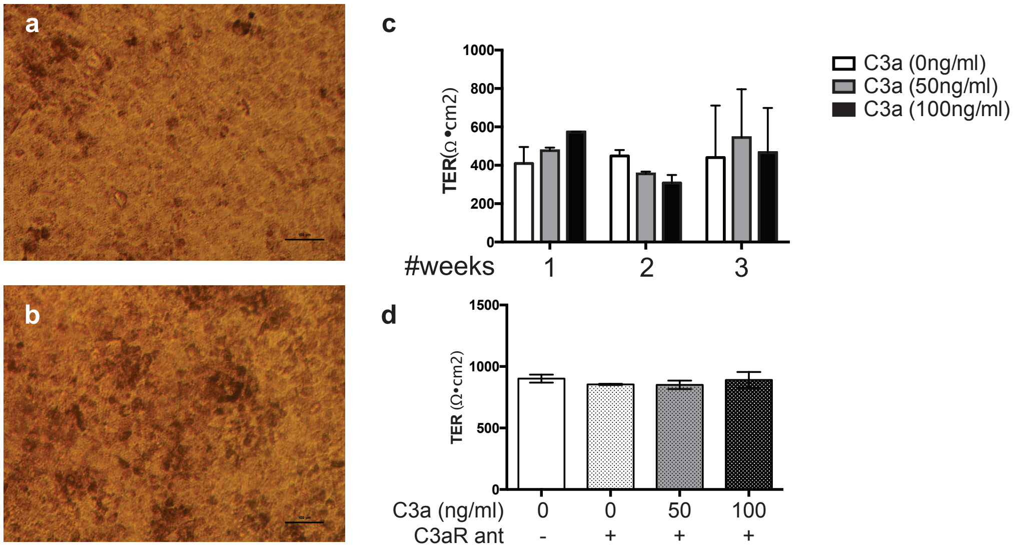

Supplementary Figure 1. Treatment with C3a and C3aR does not affect the morphology of the RPE monolayer. Brightfield microscope: representative image of hfRPE cells after 2 weeks in culture without (a) or with (b) recombinant C3a. (c) TER of hfRPE cells treated with different doses of C3a for 1, 2 or 3 weeks. (d) TER of hfRPE cells grown for 2 weeks and treated with different doses of C3a with C3aR antagonist for 2 weeks. No significant differences were found among cultures.

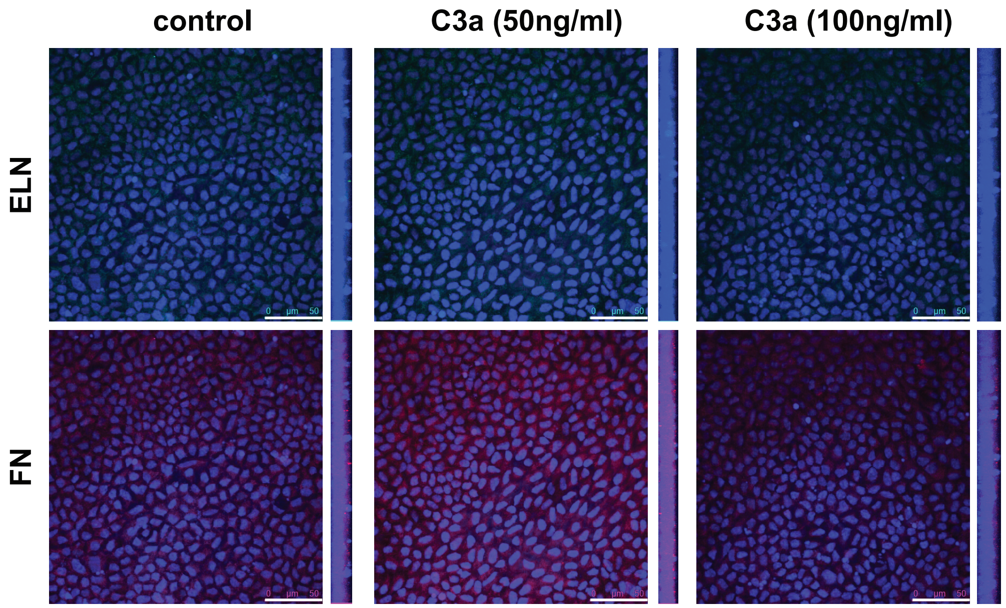

Supplementary Figure 2. Levels of elastin and fibronectin do not change with C3a treatment. Immunolabeling with antibodies for elastin (ELN) and fibronectin (FN) of hfRPE cells treated with different doses of C3a for 2 weeks. Images were taken with confocal. Orthogonal views show the negligible amount these ECM proteins underneath the RPE (left: apical, right: basal). Scale bars 50µm.

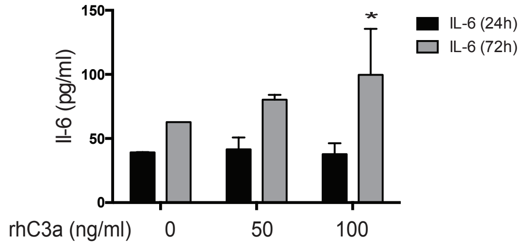

Supplementary Figure 3. IL-6 measured by ELISA in conditioned media of hfRPE cells treated with different doses of C3a for 24 (black bars) and 72 (grey bars) hours. Data represented as mean ± SD. n=6. 2-way ANOVA *$p<0.05$.
